# Validation of a Farsi version of the Early Childhood Oral Health Impact Scale (F-ECOHIS)

**DOI:** 10.1186/1472-6831-10-4

**Published:** 2010-04-06

**Authors:** Seyed-Ebrahim Jabarifar, Ali Golkari, Mohammad H IJadi, Mehdi Jafarzadeh, Parvin Khadem

**Affiliations:** 1 Isfahan Dental School, Isfahan University of Medical Sciences, Isfahan, Iran; 2 Department of Epidemiology and Public Health, University College London, London, UK; 3 Private Dental Clinic, Tehran, Iran; 4 Khorasgan Dental School, Islamic Azad University, Isfahan, Iran

## Abstract

**Background:**

The Early Childhood Oral Health Impact Scale (ECOHIS) has recently been developed to assess oral health-related quality of life (OHRQoL) of pre-school children in English speaking communities. This study aimed to translate the ECOHIS into Farsi and test its psychometric properties for use on 2- to 5-year-old children of Farsi speaking Iranian families.

**Methods:**

EHOHIS questionnaire was translated into Farsi using a standardized forward-backward linguistic translation method. Its face and content validity was tested in two small pilot studies. In the main study, a convenience sample of 260 parents of 2- to 5-year-old children in Isfahan and Tehran were invited to complete the final Farsi version of the ECOHIS (F-ECOHIS) and answer two global self-rating questions about their children's dental appearance and oral health. Association between F-ECOHIS scores and answers to the two self-rating questions, and the correlation between child (9 items) and family (4 items) sections of the F-ECOHIS were used to assess the concurrent and convergent validity of the questionnaire. Internal consistency reliability of the F-ECOHIS was tested using Cronbach's alpha coefficient test and item total and inter-item correlations. One third of participants were invited to complete the F-ECOHIS again after 2 weeks to evaluate the test-retest reliability of the questionnaire.

**Results:**

Two hundred and forty six parents were included in the main study. The association between the F-ECOHIS scores and the two self-rating questions and the correlation between its child and family sections were significant (P < 0.001). Cronbach's alpha coefficient of the F-ECOHIS and its child and family sections were 0.93, 0.89, and 0.85 respectively. Coefficients did not increase by deleting any item. The corrected item total correlation coefficient ranged from 0.52 to 0.74. The inter-item correlation coefficient ranged between 0.30 and 0.73. Seventy three parents participated in the follow up study for re-testing the questionnaire. Comparison of their test and re-test scores had a weighted kappa of 0.81 and inter-class correlation (ICC) of 0.82.

**Conclusion:**

The F-ECOHIS questionnaire was valid and reliable for assessing the OHRQoL of 2- to 5-year-old pre-school children of Farsi speaking parents.

## Background

There have been major changes in how medical and dental professionals assess health and oral health status. Health is now studied in a broader view which includes the patient's perceptions and their view of how their health affects their quality of life, rather than focusing entirely on professional judgements. This new approach requires measuring quality of life to better understand factors that contribute to the individuals' perception of health.

Oral and dental health conditions are important factors affecting the quality of life. Difficulties in speaking, smiling, kissing, eating, tasting and many other physical activities as well as psychological impacts are common outcomes of adverse oral conditions [1]. Assessing the impact of the mouth and teeth on quality of life is especially important in young children as oral health status can affect their growth, weight, socializing, self-esteem, and learning abilities. Oral and dental problems can restrict normal daily activities of both children and their parents/caregivers [1,2].

Several questionnaires have been developed to measure the impacts of oral health status on adults' quality of life. Some of them were then adapted for use on school aged children [3,4]. They are usually based on self-administered questionnaires or self-reported interviews, and are sometimes accompanied by questionnaires for parents/caregivers [5-7]. However, assessing oral health status of preschool children, and its impact on quality of life, needs a special approach. Young children have specific oral health needs. Their memory is unreliable, and they are not able to fully express themselves [8,9]. There are few questionnaires which are specifically designed to assess oral health-related quality of life (OHRQoL) in young children [5,8,10,11]. One of them, the Early Childhood Oral Health Impact Scale (ECOHIS) [11] has been specifically developed for pre-school children and has shown high degrees of success [11-14]. The ECOHIS consists of 13 questions about the impacts of oral health on child's (9 questions) and family's (4 questions) daily activities. Each question asks about frequency of an oral health-related problem and is scored from 1 (never) to 5 (very often) with a choice of "I don't know" [11].

The objective of this study was to adapt the ECOHIS for pre-school children of Farsi speaking families in Iran. Therefore, it was necessary to translate the ECOHIS into Farsi and to re-establish its psychometric properties for the geographically and culturally different population [15].

## Methods

The study consisted of a translation phase including two small pilot studies to assess the content and face validity of the instrument, a main study to assess its concurrent and convergent validity and internal reliability, and a follow up study to assess its test-retest reliability. Studies were conducted in two Iranian cities, Isfahan and Tehran. Ethical approval was obtained from the Medical Ethics Committee of Isfahan University of Medical Sciences (Ref: P/12/3/4708, Date: 26/May/2008). SPSS statistical package and a confidence interval of 95% were used for data analysis purposes.

### Translation and content and face validity

The original 13-item English version of the ECOHIS was translated into Farsi based on a linguistic translation exercise specifically standardized for translation of the health-related quality of life questionnaires [16,17]. The translation process consisted of 6 stages which included *content and face validity *assessments:

1. The original English version was translated into Farsi by two bilingual people whose first language was Farsi. One of them was a health professional, familiar with quality of life questionnaires and the other was a lay person without previous knowledge in this field. Both translators rated the difficulty of translating each item, made comments if necessary and suggested alternatives for difficult words or phrases.

2. The two translators worked with one of authors (S.E.J.) to develop a *common consensus translation (CCT)*. During this stage the readability of the questionnaire was tested [18,19]. Alternative words were used to make it easy to read even by parents with only primary school education. The group tried to use short and simple words without making the questions shorter, as a longer question would give the respondent more time to think about the question while reading it [20].

3. The CCT version was then translated back into English using two bilingual lay people whose first language was English. The back-translations were compared with the original questionnaire by two English speaking health professionals to make sure that the differences were minor and all items had the same reflections as the originals.

4. An expert committee consisting of one of the authors (A.G.), forward-translators, two other health professionals and a linguistic professional developed the *pre-final version *of the translation by considering meaning of the words and sentences, applicability of the questions, and cultural issues. Some changes were necessary for the questionnaire to be applicable to the Iranian culture and lifestyle. For example, a high number of Iranian mothers, who will be the main respondents, were housewives with no official job. Therefore, their children's illness would not result in taking time off from work (item 12), but might disrupt their normal daily activities at home or limit the time they usually spend with their other children. The expert committee worked on this stage until all members were satisfied with the semantic, idiomatic, experimental, and conceptual equivalencies [16] of the pre-final questionnaire.

5. Ten parents of 2- to 5-year-old children attending a paediatric dental practice were asked to complete the pre-final version. They were then interviewed about their views of each item. No item was rated as difficult to understand by any of parents. Three items were left blank by one or two parents, and the other 10 items were answered by all 10 parents. This showed a reasonable distribution of responses. Some parents provided comments on font size and style.

6. The final translation was developed in appropriate font and design and stage 5 was repeated with another group of ten parents. No problem was reported by parents who completed the final version of the F-ECOHIS in the second pilot study. Only two respondents had left one question blank.

### Main study assessing concurrent and convergent validity

Adults who attended four clinics in Isfahan and two clinics in Tehran during a specific period in 2009 were asked if they had a 2- to 5-year-old child. They were not necessarily seeking dental or general treatment for the child included in this study. Those who were the main caregiver of a 2- to 5-year old child, and were native Farsi speaker were invited to complete the F-ECOHIS questionnaire. The process was continued until 260 parents were invited. Those who did not give consent for the main or follow up study and those who were not aware of the child's life circumstances in a period of more than three months were excluded.

Participants completed the F-ECOHIS questionnaire and were asked about child's age and sex, reason for their attendance to the clinic, and family's socio-economic status. They were also asked to answer two global self-rating questions about: (1) their satisfaction with their child's dental appearance, and (2) their perception of child's oral health (each answered in four categories). There were a few missing answers to some items of the F-ECOHIS. A value equal to the subject's average score in child or family section, whichever the missing answer belonged to, was given to the missing answer for validity analysis purposes. "I don't know" answers were dealt with in the same way.

The reason for attendance was re-categorized into three categories of 'seeking dental treatment for the subject child', 'routine dental check up for the subject child', and 'other reasons'. The difference between F-ECOHIS scores among the three categories was assessed. The hypothesis behind this test was that those parents who were seeking dental treatment for their children were more likely to report that their children had experienced dental problems, and therefore, would report higher F-ECOHIS scores than those in other two categories. And those who brought their children for a dental check up were more likely to score higher than those attended for other reasons. Kruskal-Wallis Test was used to test the difference between the three categories as the F-ECOHIS score was not normally distributed. Furthermore, the F-ECOHIS scores of those seeking dental treatment was tested against all others using Mann-Whitney Test. These two tests were repeated after adjustments were made for child's age, sex, and city, using Poisson regression model. The association between family's socio-economic status and the F-ECOHIS score was also assessed using the Kruskal-Wallis Test before any adjustment and Poisson regression after adjustments for age, sex, and reason of attendance.

In the next step, answers to the two self-rating questions were compared against the F-ECOHIS scores to test the *concurrent (criterion-related) validity *of the scale. This is a recommended way to assess the validity of a OHRQoL questionnaire [11,21]. Kruskal-Wallis test was used for this purpose. It was hypothesized that higher level of satisfaction with dental appearance, better perceived oral health, and sum of these two (values of the two variables were added up and then re-categorized into 3 categories) were related with lower F-ECOHIS score and vice versa. Correlations between scores of the child (9 items) and family (4 items) sections of the F-ECOHIS were tested using Spearman's rank order to evaluate the *convergent (construct-related) validity *of the F-ECOHIS. The hypothesis related to this test was that the impacts of the child's oral health status on his/her life is closely related to its impacts on the family [11].

### Internal consistency reliability

*Internal consistency reliability *was tested using data from the main study by using Cronbach's alpha coefficient test and item total and inter-item correlations.

### Follow up study and test-retest reliability

A follow up study was conducted to evaluate the *test-retest reliability *of the scale. One third of those participated in the main study were randomly chosen and invited to complete the F-ECOHIS questionnaire again after 2 weeks. The degree of agreement between the two responses was tested by the weighted kappa and intra-class correlation coefficient (ICC) [22].

## Results

### Main study

Ninety five percent (N = 246) of the 260 invited parents participated in the main study. Those who did not participate either did not give consent for the main or follow up study (3%) or were unaware of the child's/family's events for a period longer than three months (2%). The average age of the children was 4 years (Table 1). Fifty seven percent of children (N = 140) were girls and 58% (N = 103) were from Isfahan. Reasons for attending the clinics were seeking dental treatment for the subject child (24%), routine dental check up for the subject child (19%), and other reasons (57%). F-ECOHIS scores ranged between 13 (the minimum possible score) and 58 (the maximum possible score was 65). The average score was 25.7 with standard deviation of 10.6. The average scores of child and family impact sections were 16.7 and 9.0 respectively (Table 2). The response rate to each item ranged from 96% to 100%.

**Table 1:** Frequency of children in each age group.

Age	Number of children	Percentage %
2 years to 2 years and 11 months	14	5.7
3 years to 3 years and 11 months	33	13.4
4 years to 4 years and 11 months	132	53.7
5 years to 5 years and 11 months	67	27.2

Total	246	100

**Table 2:** Frequency statistics of F-ECOHIS in the main study (N = 246).

	Child impact section	Family impact section	F-ECOHIS Total
Mean	16.7	9.0	25.7
Mode	13	4	21-22
Standard deviation	6.9	4.3	10.6
Minimum	9	4	13
Maximum	38	20	58

There was a significant trend in average F-ECOHIS scores among those who attended for dental treatment, those attended for a routine dental check up and those attending for other reasons (P < 0.001). Also, those who attended clinics seeking dental treatment scored significantly higher than the other two categories combined (P < 0.001). The difference exceeded 7 scores (Table 3). The associations between F-ECOHIS score and reason of attendance in the two above-mentioned tests remained significant after adjustments were made for child's age, sex, and city (Table 3). There was no significant difference between F-ECOHIS of those from different socio-economic background.

**Table 3:** Assessing association between F-ECOHIS score and participants' reason for attending clinics.

Test	Category	Number of subjects (%)	Mean F-ECOHIS score	Significance level	
				Before any adjustment	After adjustments for age, sex, and city
Reason for attendance (Two categories)				P < 0.001*	P < 0.001^†^
	Dental treatment	59 (24)	31.0		
	Dental routine check up or other reasons	187 (76)	23.6		
Reason for attendance (Three categories)				P < 0.001**	P = 0.001^†^
	Dental treatment	59 (24)	31.0		
	Dental routine check up	47 (19)	24.6		
	Other reasons	140 (57)	23.3		
Total		246 (100)	25.7	-	

*Mann-Whitney Test; **Kruskal-Wallis Test, †Poisson regression

In response to the self-rating questions, 11.4% (N = 28) of parents said they were very dissatisfied with their children's dental appearance. 12.6% (N = 31) were dissatisfied, 26% (N = 64) were satisfied, and 50% (N = 123) were very satisfied. On the other hand, 8.5% (N = 21) thought their child's oral health status was very bad, 51.2% (N = 126) thought it was bad, 25.6% (N = 63) thought it was good, and 14.6% (N = 36) thought it was very good. There was no missing or "I don't know" answer to these two questions. Answers to these two questions, separately (two tests) and combined (one test), were all significantly associated with F-ECOHIS scores (P < 0.001 for all three tests). These results showed that those parents who were less satisfied with their children's dental appearance and/or thought their children had worse oral health were more likely to give their children higher F-ECOHIS scores and vice versa. This showed that the concurrent validity of the instrument was good. In relation to convergent validity, scores of the child and family sections of the F-ECOHIS were highly correlated (P < 0.001).

The corrected item-total correlation coefficients of the F-ECOHIS, part of its internal consistency reliability measurement, ranged from 0.520 to 0.741. The lowest coefficients were related to "difficulty in pronouncing words" and "missed pre-school or school", and the highest value belonged to "trouble sleeping". The standardized Cronbach's alpha of the F-ECOHIS was 0.926. Cronbach's alpha ranged between 0.918 and 0.925 when items were deleted one by one, and did not increase by deleting any item (Table 4). The Cronbach's alpha coefficient of child and family sections of the F-ECOHIS, when tested separately, were 0.89 and 0.85 respectively. The inter-item correlation coefficients between each two of the 13 items of the F-ECOHIS ranged from 0.300 to 0.730. The weakest relationships were between "difficulty in pronouncing words" and three items of "oral/dental pain", "missed pre-school or school" and "financial impacts on family". On the other hand, the strongest correlation was found between "difficulty in drinking" and "difficulty in eating" items (Table 5). These results together suggested good internal consistency reliability for F-ECOHIS with no item being irrelevant.

**Table 4:** Internal reliability of the F-ECOHIS: Item total and alpha if item deleted statistics.

Item	Corrected item-total correlation	Cronbach's Alpha if Item Deleted
Child Impact Section		
		
Oral/dental pain	.735	.918
Difficulty in drinking	.722	.918
Difficulty in eating	.707	.919
Difficulty in pronouncing words	.520	.925
Missed pre-school or school	.521	.925
Trouble sleeping	.741	.918
Irritable or frustrated	.724	.918
Avoided smiling or laughing	.683	.920
Avoided talking	.612	.922

Family Impact Section		
		
Been upset	.640	.922
Felt guilty	.730	.918
Time off work	.729	.918
Financial impact	.684	.920

**Table 5:** Internal reliability of the F-ECOHIS: Inter-item correlation statistics.

Item number*	(1)	(2)	(3)	(4)	(5)	(6)	(7)	(8)	(9)	(10)	(11)	(12)	(13)
(1)	1.00												
(2)	.629	1.00											
(3)	.584	.729	1.00										
(4)	.305	.429	.451	1.00									
(5)	.387	.383	.393	.301	1.00								
(6)	.651	.547	.601	.359	.431	1.00							
(7)	.543	.557	.610	.407	.348	.666	1.00						
(8)	.479	.492	.473	.575	.331	.559	.615	1.00					
(9)	.390	.462	.440	.491	.321	.417	.544	.741	1.00				
(10)	.537	.480	.491	.363	.323	.474	.480	.397	.386	1.00			
(11)	.604	.478	.460	.387	.500	.571	.537	.484	.418	.657	1.00		
(12)	.632	.557	.469	.329	.499	.601	.507	.508	.419	.497	.626	1.00	
(13)	.565	.545	.492	.300	.385	.532	.511	.444	.439	.474	.585	.648	1.00

*Key to item numbers: (1): Oral/dental pain, (2): Difficulty in drinking, (3): Difficulty in eating, (4): Difficulty in pronouncing words, (5): Missing pre-school or school, (6): Trouble sleeping, (7): Irritable or frustrated, (8): Avoided smiling or laughing, (9): Avoided talking, (10): Family member been upset, (11): Family member felt guilty, (12): Family member got time off work, (13): Financial impact on family.

### Follow up study

Eighty two parents were invited for the follow up study. Nine of them refused, although they had previously agreed to participate in this part. Therefore, the response rate was 89%. Comparison between the test and retest responses revealed a weighted kappa of 0.81 and ICC of 0.82.

## Discussion

A Farsi version of the ECOHIS was developed and tested in a standardized manner. Its 13 items were all considered to be understandable and acceptable by those participated in the pilot studies. The F-ECOHIS also showed acceptable validity and reliability in the main and follow up studies.

The instrument was able to distinguish between patient groups, categorized by their reason for attendance to clinics. All inter-item correlations were positive and above the recommended level of 0.2 [23]. The corrected item-total correlations were also well above the recommended level of 0.2 [23]. Furthermore, the Cronbach's alpha coefficient of the F-ECOHIS and each of its child and family sections (0.93, 0.89, and 0.85 respectively) were above the recommended value of 0.70 [23]. These results demonstrated good internal consistency reliability for F-ECOHIS. The Cronbach's alpha values were close to those of the original English questionnaire (0.91 and 0.95 for child and family sections respectively) [11] and a Chinese version of ECOHIS (0.91 for the whole questionnaire) [24], and higher than a French version of ECOHIS (0.82, 0.79, and 0.79 for the ECOHIS, child section, and family section respectively) [13].

One might claim that the relatively high Cronbach's alpha coefficient obtained in this study was due to the large number of items in the F-ECOHIS [25] when compared with some other questionnaires used to assess the OHRQoL of children [4]. However, the Cronbach's alpha coefficient of the F-ECOHIS was compatible with those of original Child Perception Questionnaire for 8 to 10 year olds (CPQ_(8-10)_) (alpha = 0.89) [26] and its Brazilian version (alpha = 0.92) [27], which had higher number of questions. Furthermore, the Cronbach's alpha value did not decrease much when each section of the F-ECOHIS, with only 9 or 4 items, was tested separately. The alpha value of the Brazilian version of CPQ_(8-10) _decreased to 0.62 and 0.85 when divided into two sections [27]. Cronbach's alpha coefficient of the English version of the Child Oral Impacts on Daily Performances (Child-OIDP) with 8 items has been reported as 0.58 [4].

The Cronbach's alpha coefficient of the F-ECOHIS did not increase by deleting any item. This supported the idea that no item was irrelevant. Furthermore, the significant association between the F-ECOHIS scores and self-rating questions, and also, the significant correlation between the child and family sections of the F-ECOHIS confirmed good concurrent and convergent validity of the questionnaire. The ICC value of the F-ECOHIS (0.82) was close to the ICC value of the original ECOHIS (0.84) [11] and was higher than a Chinese version of ECOHIS (0.64) [24]. The ICC value of the F-ECOHIS along with its weighted kappa of 0.81 showed good agreement between test and retest results [11,28] and demonstrated acceptable level of the instrument's reproducibility.

It should be remembered that the ECOHIS and hence the F-ECOHIS are entirely based on the perceptions of parents/caregivers and their understanding of health and illness of the child. Parents of a child may have different views about their child's health from each other and from the child. The issue becomes more important when a child has been taken care of by different people, for example mother and grandmother, in different periods of life. Another issue is that this instrument evaluates the OHRQoL of the child since birth. This is an advantage because it assesses the whole life instead of a short period of life. However, assessing the whole life has two limitations: (1) the period of assessment was different from child to child based on their age, and (2) some parents were confused whether they should include impacts of teething periods. Therefore, a sentence should be included in the introduction section of the questionnaire to explain whether respondents should include difficult teething periods as adverse oral conditions.

One of the limitations of the current study was the unequal distribution of age in the main study's sample. While the data were collected on children of 4 age groups, from 2 to 5 years old, more than half of the sample was in one group: the 4 year olds. As explained in the 'Methods' section, during the sample recruitment the adults, not the children, were targeted. Data collectors asked the adults of no particular characteristics if they had any 2- to 5- year-old child. Those who answered 'yes' were invited into the study. Such an unequal age distribution was not expected and was not realized until the data was analyzed, because data were collected in six different locations. The data on age was collected in categories. A slightly better distribution might have been obtained if the age was considered as a continuous variable by asking children's date of birth instead of age in years. However, the authors did not consider the unequal age distribution a major problem, as this was a validation study and the sample size was large. Some studies on validation of OHRQoL questionnaires have had similar or even less number of subjects in some age groups due to smaller sample sizes, although the age was more equally distributed in their sample [24,27].

Considering the strengths, limitations, and results of this study, the F-ECOHIS demonstrated acceptable standards. Its psychometric properties can be reviewed while being used on larger and more diverse samples. Its appropriateness for use with children of Iranian parents who speak Farsi, but not as their first Language should be tested. The questionnaire used in this study, containing the F-ECOHIS instrument along with questions about the relationship of the respondent with the child, sex and age of the child, and the two self-rating questions is provided (see Additional file 1). Alternatively, this is available free of charge by request from the authors.

## Conclusion

The F-ECOHIS questionnaire is a valid and reliable tool to assess the OHRQoL of 2- to 5-year-old pre-school children of Farsi speaking Iranian parents.

## Competing interests

The authors declare that they have no competing interests.

## Authors' contributions

SEJ designed the study with inputs from AG. All authors were involved in translation process and field work. AG conducted the analysis and prepared the draft with inputs from SEJ. All authors read and approved the final manuscript.

## Pre-publication history

The pre-publication history for this paper can be accessed here:

http://www.biomedcentral.com/1472-6831/10/4/prepub

## Supplementary Material

Additional file 1 **F-ECOHIS_f (Word 97-2003 Document)**. F-ECOHIS instrument with child demographic questions and two self-rating questions, In Farsi Language. Click here for file
