# Supplementary material for: Validation of a Farsi version of the Early Childhood Oral Health Impact Scale (F-ECOHIS)
Source: BMC Oral Health. 2010 Apr 6;10:4. doi: 10.1186/1472-6831-10-4 (PMC2858088; doi:10.1186/1472-6831-10-4)
Supplement: Additional file 1 — F-ECOHIS_f (Word 97-2003 Document). F-ECOHIS instrument with child demographic questions and two self-rating questions, In Farsi Language. [file 1472-6831-10-4-S1.DOC]

(F-ECOHIS) پرسشنامه تأثیر سلامت دهان و دندان برکیفیت زندگی در کودکان دو تا پنج ساله

والدین گرامی

مشکلات دهـان، دندانها و فکین و درمانهای دندانـپـزشکی می تـوانـد بر سلامت و زنـدگی روزمرهَ کـودکـان

و خانواده ایشان تأ ثیر بگذارد.

لطفأ برای هر یک از سؤالات زیر، بر اساس تجربیات کودکتان یا خودتان علامـت ضربدر"**×**" را در مـربع جلوی بهترین جواب قرار دهید. تـوجه کنید که در مـوقع پاسخ به سؤالات از بـدو تـولد فـرزنـدتان تا کـنون را

در نظر بگیرید. اطلاعات این پرسشنامه محرمانه خودهد ماند. از همکاری شما صمیمانه متشکریم.

تکمـیل کنندهَ پرسشنامه: مادر پدر غیره

جنس کودک: دختر پسر

سـن کودک: ٢سال تا ٢سال و ١١ماه

٣سال تا ٣سال و ١١ماه

٤سال تا ٤سال و ١١ماه

٥سال تا ٥سال و ١١ماه

١) تا کنون کودک شما چند بار دچار **درد دندان، دهان، یا فک** شده است؟

هرگز خیلی بندرت فقط چند بار چندین بار به دفعات نمی دانم

٢) تا کنون کودک شما، به عـلت نـاراحتی های دهان و دندان یا درمانهای دندانپزشکی، چند بار در **نـوشیدن مایعات گرم یا سرد** دچار مشکل شده است؟

هرگز خیلی بندرت فقط چند بار چندین بار به دفعات نمی دانم

٣) تا کنون کودک شما، به عـلت نـاراحتی های دهان و دندان یا درمانهای دندانپزشکی، چند بار در **خوردن غـذا** دچار مشکل شده است؟

صفحه ٢

هرگز خیلی بندرت فقط چند بار چندین بار به دفعات نمی دانم

٤) تا کنون کودک شما، به عـلت نـاراحتی های دهان و دندان یا درمانهای دندانپزشکی، چند بار در **تـلفـظ لغات دچار** مشکل شده است؟

هرگز خیلی بندرت فقط چند بار چندین بار به دفعات نمی دانم

٥) تا کنون کودک شما، به عـلت نـاراحتی های دهان و دندان یا درمانهای دندانپزشکی، چند بار از **مهد کودک یا پیش دبستانی غیبت** کرده است؟

ادامه در صفحه سه

هرگز خیلی بندرت فقط چند بار چندین بار به دفعات نمی دانم

٦) تا کنون کودک شما، به عـلت نـاراحتی های دهان و دندان یا درمانهای دندانپزشکی، چند بار **بی خوابی یا کم خوابی** داشته است؟

هرگز خیلی بندرت فقط چند بار چندین بار به دفعات نمی دانم

٧) تا کنون کودک شما، به عـلت نـاراحتی های دهان و دندان یا درمانهای دندانپزشکی، چند باردچار **بیقراری یا آشفتگی** شده است؟

هرگز خیلی بندرت فقط چند بار چندین بار به دفعات نمی دانم

٨) تا کنون کودک شما، به عـلت نـاراحتی های دهان و دندان یا درمانهای دندانپزشکی، چند بار از **لبخند زدن یا خـندیدن خودداری** کرده است؟

هرگز خیلی بندرت فقط چند بار چندین بار به دفعات نمی دانم

٩) تا کنون کودک شما، به عـلت نـاراحتی های دهان و دندان یا درمانهای دندانپزشکی، چند بار از **صحبت کردن خودداری** کرده است؟

هرگز خیلی بندرت فقط چندبار چندین بار به دفعات نمی دانم

١٠) تا کنون به عـلت نـاراحتی های دهان و دندان یا درمانهای دندانپزشکی کودکتان، چند بار **شما یا دیگر اعضای خانواده نگران** شده اید؟

صفحه ٣

هرگز خیلی بندرت فقط چند بار چندین بار به دفعات نمی دانم

١١) تا کنون به عـلت نـاراحتی های دهان و دندان یا درمانهای دندانپزشکی کودکتان، چند بار **شما یا دیگر اعضای خانواده احساس تقصیر** کرده اید؟

هرگز خیلی بندرت فقط چند بار چندین بار به دفعات نمی دانم

١٢) تا کنون به عـلت نـاراحتی های دهان و دندان یا درمانهای دندانپزشکی کودکتان، چند بار **شما یا دیگر اعضای خانواده از محل کارتان مرخصی** گرفـته اید يا **از ظایف خود در خانه** باز مانده اید؟

هرگز خیلی بندرت فقط چند بار چندین بار به دفعات نمی دانم

١٣) تا کنون نـاراحتی های دهان و دندان یا درمانهای دندانپزشکی کودکتان، چند بار**فـشار اقتصادی بر خانواده شما** داشته است؟

هرگز خیلی بندرت فقط چندبار چندین بار به دفعات نمی دانم

١٤) به طور کلی چـقدر **از ظاهر دهان و دندان** کودکتان راضی هستید؟

خیلی کم کم زیاد خیلی زیاد نمی دانم

١٥) به طور کلی **وضعیت دهان و دندان** کودکتان را چگونه ارزیابی می کنید؟

خیلی بد بد خوب خیلی خوب نمی دانم

پایان
